# Supplementary material for: Supplementation of chicory root powder as an alternative to antibiotic growth promoter on gut pH, gut microflora and gut histomorphometery of male broilers
Source: PLoS One. 2021 Dec 16;16(12):e0260923. doi: 10.1371/journal.pone.0260923 (PMC8675672; doi:10.1371/journal.pone.0260923)
Supplement: Attachment — Submitted filename: Response to reviewers.docx [file pone.0260923.s002.docx]

**SUPPLEMENTATION OF CHICORY ROOT POWDER AS AN ALTERNATIVE TO ANTIBIOTIC GROWTH PROMOTER ON GUT pH, GUT MICROFLORA AND GUT HISTOMORPHOMETERY OF MALE BROILERS**

| **Reviewer comments** | **Reply by author** |
| --- | --- |
| The introduction section are given the information about the choice of the treatments. Just a small general paragraph (“The increased awareness among consumers for the poultry products without antibiotic residue encouraged the utilization of suitable alternatives for antibacterial compounds”) informs that there are “antibacterial compounds” to be used. In the research, no antibacterial compound was used, but chicory root powder was used. Moreover, what are the **biological active substances of this herb root powder**? If only a experimental plan could be built on these compounds, the approach would be more accurate. | - The anti-microbial activity of chicory root powder due to **inulin** which contains prebiotic action - Introduction was modified as per reviewer suggestion - Biological active substance in chicory powder is - Inulin |
| According to which criteria the nutritional needs of the animal are determined, the animal's house environment humidity and temperatures, the litter used, etc. such as environmental factors could be given. | - Temperature and humidity has been included in results. - The experiment was conducted in battery brooders. |
| Vitamin and mineral levels in the diet are given incorrectly. | Vitamin and mineral levels were added as per vencobb requiements. |
| It is not clear whether the nutritional composition of the basal feed was calculated or analysed. Other feed additives should not be used in feed ingredients.  In addition, the coccidiostat used in the diet is of antibiotic origin and cannot be used in finisher diets and is prohibited. Due to the danger of antibiotic residues in the tissues, the anticoccidiostat is removed from the diets of the broilers 7-15 days before slaughter. | - nutritional composition of the basal feed was calculated values - sir, we have used herbal origin coccidiostat that is **coxynil** - **moreover, we have withdrawn coccidiostats from finisher diets** |
| Since this research stands out as a preliminary experimental research, due to methodological deficiencies and flaws in terms of scientific quality. | - Chicory contains about 40 % inulin and inulin type fructans and oligofructose chains known for having prebiotic action and exert antimicrobial action. Based on this, the experiment was designed |
| I would like to state that the criticisms of the referees came to the fore, especially with the fact that performance data should be given and the number of animals was insufficient. | - Sir, performance data is going to publish in other journal. That’s why it was not included in journal |
| Ethics committee document should be provided. | - Sir, will be submitted during resubmission time |

**Reviewer #1:**

| **Reviewer comments** | **Reply by author** |
| --- | --- |
| in material and methods which requirements used to adjust feeding programme. in all feeding programm starter, grower and finisher if use prestarter used for 1st week and protein content 24%. so i see you can use starter(1-14) or follow the recommendation book of the chickens.  in table of ingredients which type of Soya bean used(44-46-48). vitamin premix try to check its composition vitamin A may be 10000000 IU. | The birds were fed with maize and soybean meal-based diets containing 2958, 3074 and 3163 kcal ME and 22.76, 21.58 and 19.68 percent crude protein, respectively during prestarter (0-14d), starter (15-28d) and finisher (28-42d) phases. We followed the breeder recommendations of vencobb.   - Soya bean used was 46. - Once agin, Vit pre mix composition was checked. It was 20000000IU only |
| Nutrient composition calculated or analyzed? | Nutrient composition on Calculated values |
| Chicory root powder composition and its content of inulin and other essential oils or contents if possible. | Sir, we have analyzed chicory root powder composition. Now, it has been included in Materials part |
| BMD composition and concentration and manufacture. | Bacitracin Methylene Disalicylate Soluble Powder 50% is a granulated biomass premix containing 150 g (activity) of bacitracin methylene disalicylate per 1 kg of product. Bacitracin Methylene Disalicylate Soluble Powder 50%  Manufacture - Zoetis |
| digital pH meter which type its model? | [Labtronics LT-50 Table-Top Microprocessor Based](https://www.indiamart.com/proddetail/microprocessor-based-ph-temp-mv-meter-graphical-display-8299665812.html) pH meter  Model No.: LT-50 |
| in table 2 or table 3 use the same groups name Chicory (0.5%) or CRP (0.5%) and not use antibiotic may use BMD. | Corrected as per reviewer suggestion |
| if possible insert dates about growth performance table and the effects of these Chicory root powder in feed intake and gain and FCR and other parameters to show effect of these treatments on broiler performance | Sorry sir, performance and other parameters are going to publish in other journal |

**Reviewer #2:**

| **Reviewer comments** | **Reply by author** |
| --- | --- |
| - SBM , add the crude protein level 44 or 46%? | Sir, 46 % level |
| 2- MEn, kcal/kg, Ca, digP , not according to the Vencobb requirement. | Feed requirements were calculated as per major and minor guides suggestion |

**Reviewer #3:**

| **Reviewer comments** | **Reply by author** |
| --- | --- |
| The author stated that in part of material and methods.One bird from each replicate was sacrificed on 42nd day of age from each treatment group. Gut (proventriculus, gizzard, duodenum and ileum) pH was recorded immediately after collection of gastro-intestinal contents from respective part of gut( . The number of samples of one sample is very, very, very small. We cannot statistically analyze and rely on these results to generalize their results and be confident in their results. The number should not be less than three samples as a minimum. | **Sir, One bird from each replicate means – 8 samples per each treatment.**  **It is adequate and sufficient for statistical analysis** |

**The figures of jejunum and Ileum were included in manuscript**

The above manuscript has been modified as per reviewer suggestion
