# Supplementary material for: Supplementation of chicory root powder as an alternative to antibiotic growth promoter on gut pH, gut microflora and gut histomorphometery of male broilers
Source: PLoS One. 2021 Dec 16;16(12):e0260923. doi: 10.1371/journal.pone.0260923 (PMC8675672; doi:10.1371/journal.pone.0260923)
Supplement: Attachment — Submitted filename: Comment PONE-D-21-14465R1.docx [file pone.0260923.s003.docx]

Title of Manuscript: Supplementation of chicory root powder as an alternative to

Antibiotic growth promoter on gut pH, gut microflora and

gut histomorphometery of male broilers

Author (s): GURRAM **et al**

**Comments**

- The manuscript is well written. The research work is interesting and good contribution in poultry.
- However, it is better if the author includes growth performance of the birds. The number of birds used in the experiment was also insufficient.
- The author stated that one bird per replica was sacrificed which is very small and statistically to rely on. The minimum sample size for statistical analysis should be three.
- Why the author focussed only on male broiler birds? Why you did not include female broiler birds?
